# Acute posterior reversible encephalopathy syndrome (PRES) in setting of interferon-beta use: case presentation with reduction of edema in 72 h after cessation of interferon-beta therapy with sub-clinical inflammation

**DOI:** 10.1186/s12883-021-02471-7

**Published:** 2021-11-10

**Authors:** Nicholas Dietz, Zarmina Mufti, Muhammed Yousaf, Randal Brown, Christopher Counts, Martin F. Bjurström, Brian J. Williams, David Robertson

**Affiliations:** 1 Department of Neurosurgery, University of Louisville, 400 Abraham Flexner Way, Louisville, KY 40202 USA; 2 Department of Neurology, 530 S Jackson St, Louisville, KY 40202 USA; 3 Department of Anesthesiology and Intensive Care, Skane University Hospital, Lund, Sweden

**Keywords:** Interferon, Multiple sclerosis, Hypertension, Posterior reversible encephalopathy syndrome, Seizure, Case report

## Abstract

**Background:**

Posterior reversible encephalopathy syndrome (PRES) represents a transient change in mental status with associated vasogenic edema of cortical and subcortical brain structures. It is often attributed to multifactorial etiology including hypertension and altered hemodynamics and disruption of vessel integrity. Patients with autoimmune disease and certain immune modulator therapies are at greater risk.

**Case presentation:**

A 54-year-old female with past medical history of well-controlled multiple sclerosis on interferon-beta since 2013, presented with witnessed tonic colonic seizure. She also was noted to demonstrate left gaze deviation and left-sided hemiparesis. MRI fluid-attenuated inversion recovery sequence showed hyperintensity of the subcortical U fibers, concentrated in the occipital, parietal lobes and frontal lobes. Systolic blood pressure was 160 mmHg on arrival. The patient was started on seizure prophylxis and Interferon beta was discontinued. The patient’s mentation, seizures and hemiapresis significantly improved in next 72 h with tight blood pressure control, and had notble improvement on MRI imaging and inflammatory markers. Lumbar puncture CSF results were devoid of infectious and autoimmune pathology.

**Conclusions:**

A middle-aged female with multiple sclerosis who was on chronic IFN-beta presented to the emergency room with a witnessed tonic-clonic seizure, with MRI T2 FLAIR imaging consistent with PRES. She had notable clinical improvement with decreased edema on imaging and improved inflammatory markers 72 h after cessation of IFN-beta therapy.

## Background

Posterior reversible encephalopathy syndrome (PRES), also known as reversible posterior leukoencephalopathy syndrome (RPLS), is characterized by an acute change in mental status with associated vasogenic edema of the parietal and occipital lobes [1]. PRES was first described in 1996 [2], with symptoms of encephalopathy (92%) [3, 4], seizures (74%) [4], hypertension (70%) [3–5], headache (26%) [3], and visual changes (20%) [3]. Classic neuroimaging findings involve cortical and subcortical posterior cerebral white matter edema best identified on T2 fluid-attenuated inversion recovery (FLAIR) imaging [2, 5–7]. While incidence is not well-reported, it is likely under-diagnosed [4]. Risk factors include female gender, middle-age, autoimmune disease, immunosuppressive therapy, history of hypertension, renal disease, and eclampsia [5, 6].

PRES is commonly reported in the setting of an acute increase in systolic blood pressure from 160 to 190 [3] mmHg, believed to result from disruption in cerebrovascular auto-regulation that leads to increased hydrostatic pressure and edema [8]. Anatomically, the sympathetic innervation predominance of the anterior circulation may be protective and explain the relative propensity for posterior circulation vasogenic edema [1]. Endothelial damage may also contribute to PRES as autoimmune, cytotoxic medications, and sepsis may increase inflammatory damage and disrupt vascular integrity leading to vascular extravasation [9, 10].

To date, a number of immunosuppressive medications identified as associated with the development of PRES, including cyclosporin A, interferon alfa and beta, intravenous immunoglobulins, erythropoietin, cisplatin, tacrolimus, and cytarabine [11]. Calcineurin inhibitors, such as cyclosporin A, are the most frequently documented medications related to PRES. Hypomagnesemia, hypocholesterolemia, the vasoactive agent endothelin, and hypertension have all been implicated in facilitating cyclosporine neurotoxicity [11]. Mastorodemos and colleagues report a case of PRES in a patient with multiple sclerosis (MS) on interferon-beta [12]. We present a case report of interferon-1beta for chronic treatment of MS implicated in acute PRES in a middle-aged female with acute hypertensive episode and seizure on presentation.

## Case presentation

**Fig. 1:**
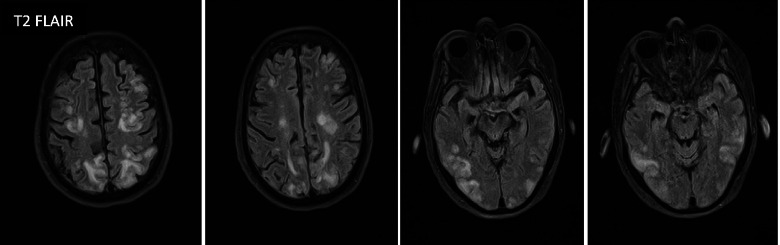
Axial T2-weighted, FLAIR images, showing extensive patchy and confluent T2/FLAIR hyperintensity of the subcortical U-fibers, most concentrated in the occipital and parietal lobes, but also visualized in the frontal lobes, bilaterally on day of admission

In March 2021, a 54-year-old female with history significant for MS diagnosed in 2013, on interferon-1beta for 7 years, squamous cell carcinoma of the neck status post neck dissection and tonsillectomy 2 weeks prior, presented to the hospital with 1 day of left sided weakness and right gaze deviation lasting 6 h, suggestive of partial onset seizure. En route to the hospital, the patient had an ictal episode consisting of bilateral arm flexion and turning to the left lasting around 15 s during which she was unresponsive. On arrival to the emergency department, the patient demonstrated postictal confusion and lethargy. There was no prior history of stroke or seizures. Patient’s exam was notable for right gaze preference, though able to track across midline, and 4/5 strength in left upper extremity, and 3/5 strength in left lower extremity with National Institutes of Health (NIH) Stroke Scale of 4 on admission. The initial concern was for stroke and patient had computed tomography angiogram (CTA) Head and Neck were unremarkable. Patient’s *laboratory values* were notable for C-reactive protein (CRP) of 12.74 mg/L, erythrocyte sedimentation rate (ESR) of 42 mm/hr. LP showed CSF remarkable only for isolated elevated protein at 61.6. Patient’s vitals were only significant elevated blood pressure at 160/68 mmHg. Urgent MRI Brain revealed extensive patchy and confluent T2/FLAIR hyperintensity of the subcortical U-fibers, most concentrated in the occipital and parietal lobes, but also visualized in the frontal lobes, bilaterally. Also, innumerable punctate foci of post contrast enhancement, with subtle cortical gradient low signal at the apical pre-and post-central gyral region suggestive of petechial hemorrhage. Overall, these findings are most consistent with posterior reversible encephalopathy syndrome (PRES) Fig. 1**.**

**Fig. 2:**
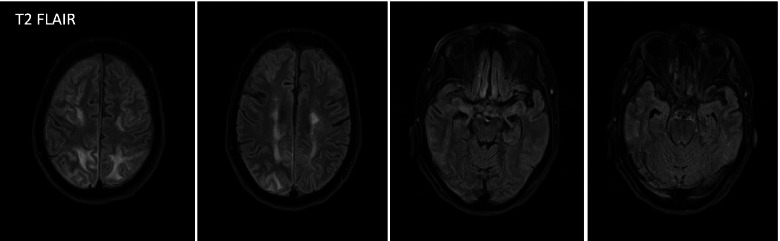
Axial T2-weighted, FLAIR images, showing almost complete resolution of the extensive patchy and confluent hyperintensities in the occipital, parietal lobes, and areas of frontal lobe 3 days later

The patient was taking beta-interferon (Extavia) every other day (QOD) 0.3 mg for MS totaling 15 doses monthly. Her home interferon therapy was discontinued due to concern of PRES secondary to interferon vs carcinomatous/paraneoplastic process. No further seizure episodes were noted during inpatient stay, which was confirmed with continuous electroencephalography (EEG) that was negative for ictal activity. Upon repeat examination on April 5th, ESR was 37 and CRP was 7.14. The EEG recording showed no evidence of ictal activity. A repeat MRI was performed 3 days later, showing improvement of the imaging features most suggestive of PRES. She was not restarted on immunomodulatory therapy after PRES diagnosis. The previously noted multifocal patchy enhancement of mostly the cortex in both cerebral hemispheres, predominantly in the bilateral parieto-occipital locations are no longer enhancing. The superimposed T2 and T2 FLAIR hyperintensities involving mostly the white matter and some of the cortex in these regions were minimally improved. However, there was new T2 FLAIR signal in the sulci diffusely in the bilateral cerebral convexities predominantly adjacent to the areas of T2 FLAIR hyperintensity likely related to the vascular hemodynamics of PRES perhaps with protein leakage, Fig. 2.

## Discussion and conclusions

Posterior reversible encephalopathy syndrome is a recently described (1996) and likely underreported phenomenon with complex etiology secondary to alterations in hemodynamics, osmolality, inflammation and endothelial dysregulation [1]. It may be acutely exacerbated by transient rise in blood pressure and is often seen in the setting of autoimmune disease, immunosuppressive drugs, and infection [3, 13]. Seizures on initial presentation is common, and would raise index of suspicion for PRES in middle-aged females with acute-onset encephalopathy and uncontrolled systolic blood pressure [3]. MRI brain without contrast should be ordered to assess FLAIR sequence with attention to parietal and occipital lobes and cerebellar hemispheres. Protein, albumin, and nutritional status may also be assessed given its concurrent presentation with PRES. [14] Management includes blood pressure control, and an attempt to remove offending agents that may contribute to endothelial damage such as immune-modulating therapies with assessment of risk benefit ratio regarding treatment.

The present case involves a middle-aged female with 7-year history of MS using IFN-beta, an established and generally well-tolerated disease-modifying therapy that has demonstrated consistent efficacy in MS populations [15, 16]. A recent report has also identified IFN-beta in PRES. [12] Studies show that IFN-beta may induce a thrombotic microangiopathy with dose-dependent toxic effect on cerebral microvasculature [17]. Moreover, IFN-beta stimulates the vasoconstriction cascade, causing fibromuscular intimal proliferation of arterioles and arteries, leading to hypertension and ultimately PRES. [18] Chronic IFN-beta treatment may also disrupt the blood brain barrier integrity, exacerbating likelihood of protein leakage and vasogenic edema. The MRI revealed T2 FLAIR signals in the sulci in the bilateral cerebral convexities, which are likely related to the vascular and potentially osmotic hemodynamic changes. Notably, the patient’s albumin was low at 2.0 and total protein was 4.9, noted to be low in patients with PRES. [14] Protein improved to 5.2 and albumin remained low at 2.2 at time of second MRI Brain that showed significant improvement in FLAIR signal abnormalities. LDH has been previously reported to be associated with larger distribution of edema [19]. The patient’s LDH was within normal limits at 165. The patient had no other active signs of infection or fever, but was noted to have an elevated ESR and CRP on admission. Both inflammatory marker values were observed to decrease in 72 h. To the extent that inflammatory cytokines may contribute to endothelial dysfunction [20], ESR and CRP may be important to monitor during recovery. ESR has been previously associated with poorer prognosis in PRES [21], but little investigation on auto-immune flare or sub-clinical inflammation has been conducted. Endothelial dysfunction due to potential cytotoxicity of IFN-beta and inflammation. Given that 30% of patients with PRES are normotensive at time of diagnosis, some question the degree to which hypertension may contribute [22]. Perhaps more attention should be paid to oncotic properties of low albumin and protein in the setting of PRES.

Evidence of frontal vasogenic edema was also observed in addition to the typical presentation of parietal and occipital cortical edema. In a large cohort analysis of PRES neuroimaging, including 114 patients with MRI, Bartynski and Boardman demonstrate frontal edema in 68% of patients, parietal and occipital edema in 98% of patients, 40% in temporal lobes, and 30% in the cerebellum [23]. From the lumbar puncture on admission, protein was elevated to 61.6 on admission, consistent with previous reports of PRES that show elevation of CSF protein in up to 70% of patients [24]. Resolution has most often been described to be radiographically evident after weeks despite clinical improvement occurring earlier [5, 8]. Further, acute reduction in characteristic vasogenic edema may be noted for particularly acutely resolving FLAIR signal.

To our knowledge, little investigation has been conducted related to oncotic contribution to PRES, acute resolution, and frontal vasogenic edema in setting of IFN-beta. The present case supports recently described phenomenon of IFN-beta contribution to PRES and confirms hypertension, hypoproteinemia, elevated inflammatory markers at time of presentation, indicating complex mechanisms involving alterations in hemodynamics, osmolality, and local inflammation in characteristic locales of the brain. Future studies may investigate the degree to which auto-immune flares or sub-clinical inflammation are concurrent with PRES, as well as nutritional status, low serum protein and albumin may play a role to contribute to a likely multifactorial process in the development of PRES.

## Conclusions

A middle-aged female with multiple sclerosis who was on chronic IFN-beta presented to the emergency room with a witnessed tonic-clonic seizure, with MRI T2 FLAIR imaging consistent with PRES. She had notable clinical improvement with decreased edema on imaging and improved inflammatory markers 72 h after cessation of IFN-beta therapy.

## Data Availability

Additional images available through request to corresponding author.

## Abbreviations

NIH: National Institutes of Health
PRES: Posterior reversible encephalopathy syndrome
MS: Multiple sclerosis
EEG: Electroencephalography
RPLS: Reversible posterior leukoencephalopathy syndrome
CTA: Computed tomography angiogram
CRP: C-reactive protein
ESR: Erythrocyte sedimentation rate
FLAIR: Fluid-attenuated inversion recovery
